# Proton pump inhibitors and dementia: what association?

**DOI:** 10.1590/1980-5764-DN-2022-0048

**Published:** 2023-05-29

**Authors:** Catarina Caetano, Marta Veloso, Susana Borda

**Affiliations:** 1 Unidade de Saúde Familiar Delta, Administração Regional de Saúde de Lisboa e Vale do Tejo, Oeiras, Lisboa, Portugal.

**Keywords:** Proton Pump Inhibitors, Dementia, Cognitive Dysfunction, Inibidores da Bomba de Prótons, Demência, Disfunção Cognitiva

## Abstract

Several recent studies have suggested an association between proton pump inhibitors and the development of cognitive changes.

**Objective:**

To assess the existence of this association.

**Methods:**

A comprehensive literature search was conducted including guidelines, meta-analyses, systematic reviews, observational and experimental studies published between February 2011 and February 2021.

**Results:**

The initial research identified 393 articles, 28 of which were included: 8 systematic reviews, 1 clinical trial, 15 observational studies, 3 case-control studies, and 1 cross-sectional observational study.

**Conclusions:**

Most studies classified with the highest level of evidence found no statistically significant association between the use of proton pump inhibitors and the development of cognitive impairment or dementia.

## RESUMO.

Nos últimos anos, têm sido publicados vários estudos que indicam uma associação entre o uso de inibidores da bomba de protões e o desenvolvimento de alterações cognitivas.

**Objetivo:**

Avaliar a existência desta associação.

**Métodos:**

Foi realizada uma pesquisa bibliográfica abrangente, incluindo diretrizes, meta-análises, revisões sistemáticas, estudos experimentais e observacionais publicados entre fevereiro de 2011 e de 2021.

**Resultados:**

A pesquisa inicial identificou 393 artigos, dos quais 28 foram incluídos: 8 revisões sistemáticas, 1 ensaio clínico, 15 estudos observacionais, 3 estudos de caso-controle e 1 estudo observacional transversal.

**Conclusões:**

A maioria dos estudos com o maior nível de evidência não encontrou uma associação estatisticamente significativa entre o uso de inibidores da bomba de protões e o desenvolvimento de défice cognitivo ou demência.

## INTRODUCTION

Proton pump inhibitors (PPIs) have been central to the management of acid-related upper gastrointestinal disorders for the past three decades^1^.

PPIs are among the most commonly prescribed drugs worldwide. Up to 1 in 5 older adults takes PPIs – and frequently – on a long-term basis^2^.

Various studies worldwide have pointed to the inappropriate prescription of these drugs, either in excessive dosage, inappropriate prolonged duration, or in the absence of medical recommendation^3–5^.

Along with the generalization of its use, there has been a growing concern about its adverse effects, widely disseminated through the media. Published scientific evidence on the safety profile of these drugs supports an association between long-term use of PPIs and an increased risk of developing acute and chronic kidney disease, Clostridium difficile infection, community-acquired pneumonia, bone fractures, hypomagnesemia, vitamin B12 deficiency, among others^6–9^.

These effects are more pronounced among the elderly, for which the American Geriatrics Society Beers Criteria, updated in 2019, advise against the use of PPIs for more than 8 weeks, except in situations of erosive esophagitis, Barrett esophagus, hypersecretory pathology or demonstrated need for treatment maintenance^10^.

In recent years, some studies have suggested an association between the use of PPIs and the development of cognitive impairment^11–13^.

Several mechanisms have been proposed to explain the possible association between PPIs use and the development of dementia^14–17^. One of the mechanisms focuses on the decrease in vitamin B12, which has been associated with cognitive decline and neurological damage^18^, probably due to decreased synthesis of deoxyribonucleic acid and homocysteine neurotoxicity^19,20^. There is evidence that PPIs (e.g. lansoprazole and omeprazole) cross the blood-brain barrier; therefore, several intracerebral mechanisms have been studied^21,22^. A possible factor for the effect of PPIs on cognition is through direct interaction with brain enzymes. A recent study by Badiola et al. found that PPIs, such as lansoprazole, increase levels of amyloid beta peptide (Aβ) in an amyloid cell model and in the mouse brain^23^. Aβ peptides are one of the main pathological signs of Alzheimer's disease and are also cytotoxic to endothelial cells^14^. Another mechanism described for the increase in Aβ peptide deposits by PPIs refers to the possible modulation of their degradation by lysosomes in microglia, since this process is pH-dependent and induced by lysosome acidification. This acidification has been described as mediated by the vacuolar proton pump adenosine triphosphatase and it is thought that PPIs will have an inhibitory action on these and may contribute to the inhibition of Aβ degradation and thus increase its deposit^24–26^.

This review aimed to assess whether there is an association between the use of PPIs and the development of cognitive impairment or dementia, according to the currently available scientific evidence.

## METHODS

The authors performed a literature search of meta-analyses, systematic reviews, randomized controlled trials, cohort studies, case-control studies, and guidelines published in the following databases: PubMed, Cochrane Library, Database of Abstracts of Reviews of Effects, Guidelines Finder, Canadian Medical Association Infobase e National Guidelines Clearinghouse.

We used the following MeSH terms: PPI, PPIs, proton pump inhibitors, omeprazole, pantoprazole, esomeprazole, lansoprazole or rabeprazole and cognitive impairment or dementia.

Articles published between 02/18/2011 and 02/18/2021, in English, Portuguese or Spanish, that met the following criteria were included in the review:

- Population: adults without a previous diagnosis of mild cognitive impairment or dementia;
- Intervention: use of PPIs;
- Control: placebo, H2 receptor antagonists, or no drug; and
- Outcome: development of mild cognitive impairment or dementia. Repeated articles and those that did not meet the eligibility criteria and purpose of the review were excluded.

For article selection, the authors proceeded to three phases of exclusion: regarding the title, level of evidence classification of each article, and strength of the abstract. Afterwards, the full article was read. Each one was read by two authors, resorting to the third in situations of disagreement between the first two.

The methods for the diagnosis of dementia were heterogeneous. In some studies, the diagnosis of dementia was based on cognitive tests (e.g., Mini-Mental State Examination [MMSE], Clock Drawing Test [CDT] or Abbreviated Mental Test [7-Minute Screen]), brain imaging (computed tomography [CT], magnetic resonance imaging [MRI], or single-photon emission computed tomography [SPECT]), and dementia symptoms. Often the diagnosis was confirmed by a board-certified psychiatrist or neurologist, based on the criteria of Diagnostic and Statistical Manual of Mental Disorders (DMS)-IV. In other studies, the authors assumed the diagnosis as documented based on the International Classification of Diseases (ICD) codes or prescription records for medication for treating dementia.

The association of dementia and use of PPIs could have been assessed as categorical variables, such as adjusted hazard ratio (HR) and adjusted odds ratio (OR) or relative risk (RR).

Some studies used adjusted ratios (i.e., those that adjusted for most factors).

Strength of Recommendation Taxonomy (SORT) system was used.

## RESULTS

**Figure 1:**
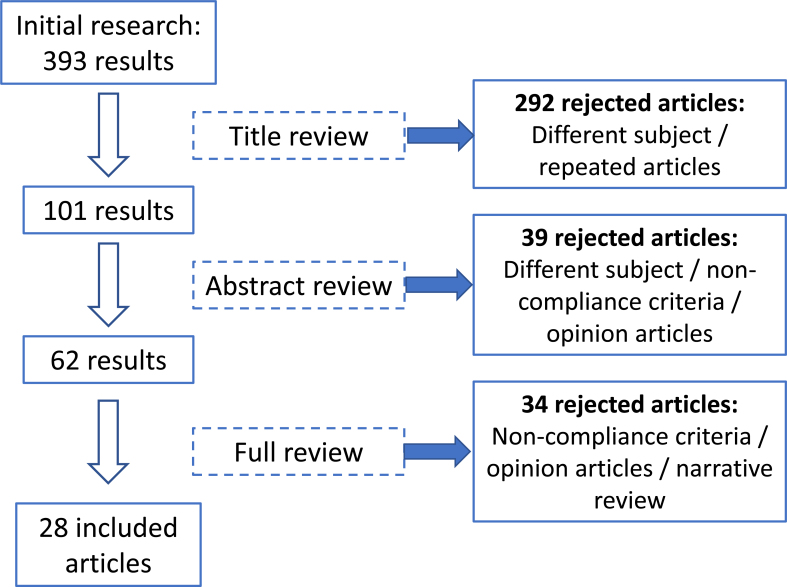
Article selection process.

From the initial search, 393 articles were obtained. After the selection process (Figure 1), 28 articles were included in the review: 8 systematic reviews (7 of them with meta-analysis), 1 randomized clinical trial, 15 cohort studies, 3 case-control studies, and 1 cross-sectional observational study.

### Clinical trials

A single randomized clinical trial was identified, which compared the use of pantoprazole 40 mg versus (vs.) placebo in patients with atherosclerotic disease (Table 1)^27^. The group of patients using pantoprazole presented an OR of 1.20 and 95% confidence interval (CI) 0.81–1.78 for the development of dementia, without statistical significance^27^.

**Table 1:** Clinical trials.

Author, year	Type of study	Sample size	Intervention	Outcome	Results	Conclusion	LE
Moayyedi et al.^27^, 2019	Multicenter double-blind randomized controlled trial	17,598	Pantoprazole 40 mg vs. placebo in patients with atherosclerotic disease	Secondary: Dementia diagnosis in the follow-up period (3 years)	OR=1.2; 95%CI (0.81–1.78) (p=0.36)	No association	I

Abbreviations: LE: level of evidence; OR: odds ratio; CI: confidence interval; p: p-value.

### Observational studies

#### Longitudinal studies

The literature search yielded six prospective and nine retrospective cohort studies (Table 2)^14,15,16,27–42^. Two of the prospective studies^14,15^ found an increased risk of developing dementia associated with PPI use, both conducted in Germany. Haenisch et al.^14^ evaluated the effect of using any PPI compared with no use of these drugs in a population aged 75 years and over, with a HR of 1.38 for all-cause dementia and 1.44 for Alzheimer's disease.

**Table 2:** Observational studies.

	LE	I	I	II	I	I	I	II		II	II
	Conclusion	Increased risk	Increased risk	Risk reduction	No association	No association	No association	Increased risk		No association	No association
Observational studies	Results	ACD: HR=1.38; 95%CI (1.04–1.8); AD: HR=1.44; 95%CI (1.01–2.06)	HR=1.44; 95%CI (1.36–1.52) (p<0.001)	From normal cognition: HR=0.78; 95%CI (0.66–0.93) (p=0.005)Progression from MCI to Dementia: HR=0.82; 95%CI (0.69–0.98) (p=0.03)	ACD: OR=1.13 (p=0.67)AD: OR=1.11 (p=0.77). Cumulative doses of: 365 TSDDs, 1,095 TSDDs and 1,825 TSDDs: HRs without statistical significance.	Mean score differences from 0.00 to −0.06 points for periods of PPI use between 1 to 14 years. p≥0.84	HR=0.99, 95%CI (0.70–1.37)	Dementia diagnosis: aSR=1.21;95%CI (1.16–1.27). Use of anti-dementia drugs: aSR=1.38 95%CI (1.28–1.48)		Elderly PPI users vs. non-users: difference of −1.22 points, 95%CI (−3.73–1.29) Individuals 46-67 years old PPI users vs. non-users: difference of 0.94 points, 95%CI (−1.63–3.50)	Prolonged use of PPIs: HR=0.99, 95%CI (0.93–1.17) Intermittent use of PPIs: HR=0.91, 95%CI (0.76–1.09)
	Outcome	Diagnosis of all-cause dementia or Alzheimer's disease	Dementia diagnosis	Diagnosis of LBD (Lewy Bodies Dementia)Dementia from normal cognition or diagnosis of dementia in patients with LBD.	Diagnosis of all-cause dementia or Alzheimer's disease	Neurocognitive assessment performance	Dementia diagnosis	Dementia diagnosis or prescription of anti-dementia drugs		Neurocognitive assessment performance	Dementia diagnosis
	Intervention	PPIs use	PPIs use	PPIs use	PPIs use (cumulative dose)	PPIs use	Cumulative dose PPIs vs. H2 blockers	PPIs use		PPIs use	PPIs use
	Sample	3,076	73,679	10,486	3,484	13,864	70,529	1,000,000		7,878	10,533
	Type of study	Prospective cohort	Prospective cohort	Retrospective cohort	Prospective cohort	Prospective cohort	Prospective cohort	Retrospective cohort		Prospective cohort	Retrospective cohort
	Author, Year	Haenisch et al.^14^, 2015	Gomm et al.^15^, 2016	Goldstein et al.^35^, 2017	Gray et al.^28^, 2017	Lochhead et al.^27^, 2017	Hwang et al.^30^, 2018	Park et al.^31^, 2018		Wod et al.^29^, 2018	Huang et al.^37^, 2019
Observational studies	LE	II	II	II	II	II	II	II	II	II	II
	Conclusion	No association	Increased risk	Increased risk	Risk reduction	Increased risk	No association	No association	Risk reduction	No association	No association
	Results	IRR=1.01;95%CI (0.96–1.06)	OR=1.55 (p<0.001)	aHR=1.42;95%CI (1.07–1.84) Association between cumulative dose and risk of dementia with statistical significance (p-trend<0.001)	HR=0.67, 95%CI (0.65–0.67)(p<0.01)	AD: aOR=1.06;95%CI (0.93–1.21)nAD: aOR=1.20, 95%CI (1.05–1.37)(p=0.007). AD for high dose PPI: aOR=1.20; 95%CI (0.91–1.61)nAD for high dose PPI: aOR=0.95, 95%CI (0.74–1.22)	aHR=0.72;95%CI (0.51–1.03)	aOR=1,0; 95%CI (0,40–2,73)	OR=0.93, CI95% (0.90–0.97)(p=0.0008)	aOR=1.01;95%CI (0.97–1.06)	AD: aOR=0.88; 95%CI (0.80–0.97)VV: aOR=1.18, 95%CI (1.04–1.33)
	Outcome	Dementia diagnosis	Dementia diagnosis or prescription of anti-dementia drugs	Dementia diagnosis	Dementia diagnosis	Diagnosis of Alzheimer's disease or non-Alzheimer dementia	Dementia diagnosis	Dementia diagnosis	Dementia diagnosis	Alzheimer's Disease Diagnosis	Development of Alzheimer's Disease or Vascular Dementia
	Intervention	PPIs use	PPIs use	PPIs use (cumulative dose)	PPIs use	PPIs use	PPIs use (and H2 blockers)	Continuous PPIs’ use	PPIs use	PPIs use (cumulative dose)	PPIs use
	Sample	304,753	23,656	62,574	315,078	135,722	92,773	7,8 billion	23,912 (1:1)	353,576 (1:4)	41,029
	Type of study	Retrospective cohort	Retrospective cohort	Retrospective cohort	Retrospective cohort	Retrospective cohort	Retrospective cohort	Cross-sectional	Case control	Case control	Case control
	Author, Year	Park et al.^36^, 2019	Welu et al.^32^, 2019	Chen et al.^33^, 2020	Cooksey et al.^34^, 2020	Torres-Bondia et al.^16^, 2020	Wu et a.l^38^, 2020	Ma et al.^42^, 2020	Booker et al.^41^, 2016	Taipale et al.^40^, 2017	Imfeld et al.^39^, 2018

Abbreviations: LE: level of evidence; ACD: all-cause dementia; HR: hazard ratio; AD: Alzheimer's dementia; CI: confidence interval; p: p-value; MCI: Mild Cognitive Impairment; TSDDS: Total standardized daily doses; aSR: adjusted sequence ratio; PPI: Proton pump inhibitors; IRR: incidence rate ratio; aHR: adjusted hazard ratio; aOR; adjusted odds ratio; RR: relative risk; nAD: non-Alzheimer dementia.

For seven years, Gomm et al.^15^ followed a cohort of individuals under regular use of PPIs with at least one prescription per quarter and also concluded the existence of this slight increase in risk (HR 1.44) compared with non-use.

The remaining prospective studies did not demonstrate the existence of this association, either in comparison with placebo or with H2-antagonists^28–30,43^.

In Denmark, Wod et al.^29^ studied two cohorts of twins in different age groups (middle-aged and elderly), comparing PPI users and non-users, and had no statistically significant difference in cognitive decline between groups in both cohorts. When studying the effect of PPIs over the time of use^28^ or adjusted for cumulative dose^30,43^, there was also no increase in the risk of developing dementia.

Regarding retrospective studies, four of them^16,31–33^ found an association between the use of these drugs and cognitive decline. In Spain, Torres-Bondia et al.^16^ found a slight increase in the risk of non-Alzheimer dementia compared with PPI non-users (adjusted odds ratio [aOR] 1.20), with no dose-dependent effect, which was not observed for Alzheimer's disease. In the United States of America (USA), Welu et al.^32^ showed a 51% increase in the risk of developing dementia in a cohort of more than 23,000 war veterans with PPI consumption for more than 30 days compared to an equal number of veterans who had never been prescribed PPIs, although without correlation to the duration of the treatment or cumulative dose. A South Korean population cohort with about 1 million people was used for a study^31^ that found an increased risk of developing dementia in PPI users (adjusted sequence ratio [aSR] of 1.21) after 3 years of use, which was more pronounced with omeprazole (aSR 1.24). In this study, different latency times of 1, 2, and 3 years were applied and the results showed that the relative risk decreased with the rise of latency time: adjusted incidence rate ratio (aIRR) of 1.13, 1.02, and 0.89, respectively; without application of latency time, the aIRR was 1.27. A study in Taiwan on people over 65 years of age, showed an increase in this risk (aHR 1.42; 95%CI 1.07–1.84), in a dose-dependent manner^33^. In contrast, two retrospective studies by Cooksey et al.^34^(in Scotland) and Goldstein et al.^35^ (in the USA) showed a decrease of about 30% in the risk of dementia in PPI users (HR 0.67 and 0.78, respectively), either with regular or intermittent consumption of these drugs. Park et al.^36^ evaluated the risk of dementia in PPI users compared to anti-H2 users, this time not verifying the harmful effect associated with the former, contradicting their own findings published in 2018. The remaining retrospective studies considered in this research did not show an increased risk, either in continuous or intermittent use^37^ or in comparison with anti-H2^38^.

### Case control

The results of our search included three case-control studies. Imfeld et al. studied the development of Alzheimer's dementia (AD) and vascular dementia (VD) associated with prolonged PPI use in individuals over 65 years of age, and found no increased risk for either of these conditions compared with non-users (aOR 0.85 and 0.90, respectively), which remained the same when the analysis was done for each of the different PPIs or for their combined use^39^. Taipale et al. studied the development of Alzheimer's dementia associated with PPI use with 3-year lag window applied between exposure and outcome (aOR 1.03; 95%CI 1.00–1.05) compared with no use, with higher doses use (≥1.5 defined daily doses per day; aOR 1.03; 95%CI 0.92–1.14) and with longer duration of use (≥3 years of use; aOR 0.99; 95%CI 0.94–1.04), revealing no increased risk^40^. Another case-control study, carried out in Germany, which main outcome was the development of dementia dependent on predefined risk factors, concluded that there may be a modest protective effect of PPIs on the development of dementia (HR 0.93; 95%CI 0.90–0.97), along with statins and antihypertensives (Table 2)^41^.

### Cross-sectional study

The only observational cross-sectional study obtained encompassed more than 7 billion surveys conducted in North American patients and did not demonstrate the existence of any association between PPI use and the development of dementia (Table 2)^42^.

### Systematic reviews and meta-analyses

The search resulted in eight systematic reviews, seven of them comprising meta-analysis (Table 3)^12,13,17,44–48^. A systematic review published in 2017 included 11 studies with different methodologies and great heterogeneity, suggesting an increased risk of dementia with PPIs, but meta-analysis was not performed^13^. One of the identified meta-analyses encompassed six cohort studies and found a slightly increased risk of dementia as a result of PPI use, with a HR of 1.29, and 95%CI 1.12–1.49^44^.

**Table 3:** Systematic reviews.

Systematic reviews						
Author, Year	Type of study	Sample	Studies included	Results	Conclusion	LE
Batchelor et al.^13^, 2017	Systematic reviews	101,616	One experimental study and 10 observational studies (4 cohort, 1 case-control, 1 cross-sectional, 1 case series, and 3 case reports)	No meta-analysis	Increased risk	II
Hussain et al.^17^, 2020	Systematic review and meta-analysis	618,911	12 observational studies (8 cohort and 4 case-control)	RR=1.05, 95%CI 0.96–1.15	No association	I
Li et al.^12^, 2019	Systematic review and meta-analysis	106,599	6 cohort studies	RR=1.23; 95%CI 0.90–1.67Follow-up < 5 years: RR=1.62, 95%CI 1.40–1.86 Follow-up > 5years: RR=0.98, 95%CI 0.75–1.27	No association	I
Song et al.^47^, 2019	Systematic review and meta-analysis	642,305	10 observational studies (5 cohort, 4 case control, and 1 cross-sectional)	ACD: HR=1.04, 95%CI 0.92–1.15 AD: HR 0.96, 95%CI 0.83–1.09	No association	I
Zhang et al.^44^, 2020	Systematic review and meta-analysis	166,146	6 cohort studies	HR=1.29, 95%CI 1.12–1.49	Increased risk	I
Desai et al.^46^, 2020	Systematic review and meta-analysis	308,249	One experimental study and 5 prospective observational	ACD: HR=1.16, 95%CI 0.86–1.47	No association	I
Khan et al.^45^, 2020	Systematic review and meta-analysis	642,949	11 observational studies (6 cohort, 4 case control, and 1 cross-sectional)	ACD: HR=1.10; OR=1.03.AD: HR=1.06; OR=0.96	No association	I
Zhang et al.^48^, 2020	Systematic review and meta-analysis	371,951	10 observational studies	OR=0.87; 95%CI 0.62–1.22	No association	II

Abbreviations: RR: relative risk; CI: confidence interval; ACD: all-cause dementia; AD: Alzheimer's dementia; HR: hazard ratio; OR: odds ratio.

There is some overlap of studies included in meta-analyses, notably all cohorts included in Zhang's et al.^44^ meta-analysis are covered by Khan et al.^45^ meta-analysis.

None of the six remaining meta-analyses in review excluded in this research presented evidence to support the association between PPIs and dementia^12,17,45–48^. Among the most recent ones, the review by Khan et al.^45^, which included 11 observational studies with 642,949 individuals, found a HR for dementia from all-causes of 1.11 and 95%CI 0.88–1.37, and for Alzheimer's disease a 95%CI 0.72–1.55.

## DISCUSSION

Overall, this evidence-based review identified nine studies classified as level of evidence I (five meta-analyses, three observational studies, and one randomized clinical trial) and eight studies classified as level of evidence II (one systematic review, four longitudinal observational, one cross-sectional, and two case-control studies) that found no statistically significant evidence of a relationship between PPI use and the development of dementia. Three level of evidence I studies (one meta-analysis and two longitudinal observational studies) found an association between PPI use and dementia. In the study by Haenisch et al.^14^, information about the pattern of drug use was not consistently available. In the meta-analysis by Zhang et al.^44^, only cohort studies were included and there was substantial heterogeneity between studies; different indicators (OR and HR) were included as measures of similar effect, which constitutes a bias. In these studies, the most expressive measure of effect was a HR of 1.44, 95%CI 1.36–1.52 (p-value[p]<0 .001) which was obtained in a prospective cohort study in a population aged 75 years and over^15^. Five studies with level of evidence II found an increased risk (one systematic review without meta-analysis and four longitudinal observational studies), where the highest risk measure was an OR of 1.55^32^. The study by Batchelor et al.^13^ had several limitations, namely data heterogeneity, variability in study designs, and clinical diversity, with an important risk of bias.

The study by Park et al.^36^ highlighted the importance of applying a window time after the start of PPIs, during which cases of dementia may arise in patients without a previous diagnosis, although without an etiological relationship with the drug, thus reducing the protopathic bias. The decrease in IRR with increasing time window weakens the evidence for the association between PPI use and dementia. In three studies with level of evidence II^36,37,41^ there was a decrease in the risk of developing dementia, with the lowest HR recorded 0.67, 95%CI 0.65–0.67 (p<0.01) and resulting from a retrospective cohort study, where it was not possible to assess the duration of use or dosage of PPI^34^.

Regarding the strengths of this review, the expressive sample size of the included studies stands out, comprising data from different countries and continents. The selected outcome included cognitive impairment and dementia of all etiologies, not limited to Alzheimer's disease. Only level of evidence I and II studies were included, with most of the more robust ones were classified as level I.

As the main limitation, we highlight the great heterogeneity among the results, which are somewhat contradicting. Only one experimental study was identified, with a predominance of observational studies. Other limitations are the lack of methodological consistency of the intervention (dose, duration, pattern of intake, confirmation of treatment adherence) and the outcome (clinical diagnosis, neuropsychological tests, prescription of antidementia drugs), and the fact that it was not considered a window time in most studies. Additional research is needed because there is biological evidence that PPIs could affect the brain and increase the risk of dementia and AD.

In conclusion, although the available evidence is discordant, most level of evidence I studies have not found a statistically significant association between PPI use and the development of dementia or cognitive impairment. Therefore, we believe further studies on this topic are needed, particularly randomized clinical trials.

## References

[1] Katz PO, Gerson LB, Vela MF (2013). Guidelines for the diagnosis and management of gastroesophageal reflux disease. Am J Gastroenterol.

[2] Qato DM, Wilder J, Schumm LP, Gillet V, Alexander GC (2016). Changes in prescription and over-the-counter medication and dietary supplement use among older adults in the United States, 2005 vs 2011. JAMA Intern Med.

[3] Batuwitage BT, Kingham JGC, Morgan NE, Bartlett RL (2007). Inappropriate prescribing of proton pump inhibitors in primary care. Postgrad Med J.

[4] Matuz M, Benkő R, Engi Z, Schváb K, Doró P, Viola R (2020). Use of proton pump inhibitors in hungary: mixed-method study to reveal scale and characteristics. Front Pharmacol.

[5] Savarino V, Marabotto E, Zentilin P, Furnari M, Bodini G, Maria C (2018). The appropriate use of proton-pump inhibitors. Minerva Med.

[6] Jaynes M, Kumar AB (2018). The risks of long-term use of proton pump inhibitors: a critical review. Ther Adv Drug Saf.

[7] Yang YX, Lewis JD, Epstein S, Metz DC (2006). Long-term proton pump inhibitor therapy and risk of hip fracture. JAMA.

[8] Lambert AA, Lam JO, Paik JJ, Ugarte-Gil C, Drummond MB, Crowell TA (2015). Risk of community-acquired pneumonia with outpatient proton-pump inhibitor therapy: a systematic review and meta-analysis. PLoS One.

[9] Poly TN, Islam MM, Yang HC, Wu CC, Li YCJ (2019). Proton pump inhibitors and risk of hip fracture: a meta-analysis of observational studies. Osteoporos Int.

[10] American Geriatrics Society Beers Criteria^®^ Update Expert Panel (2019). J Am Geriatr Soc.

[11] Fallahzadeh MK, Haghighi AB, Namazi MR (2010). Proton pump inhibitors: predisposers to Alzheimer disease?. J Clin Pharm Ther.

[12] Li M, Luo Z, Yu S, Tang Z (2019). Proton pump inhibitor use and risk of dementia: systematic review and meta-analysis. Medicine (Baltimore).

[13] Batchelor R, Gilmartin JFM, Kemp W, Hopper I, Liew D (2017). Dementia, cognitive impairment and proton pump inhibitor therapy: a systematic review. J Gastroenterol Hepatol.

[14] Haenisch B, von Holt K, Wiese B, Prokein J, Lange C, Ernst A (2015). Risk of dementia in elderly patients with the use of proton pump inhibitors. Eur Arch Psychiatry Clin Neurosci.

[15] Gomm W, von Holt K, Thomé F, Broich K, Maier W, Fink A (2016). Association of proton pump inhibitors with risk of dementia: a pharmacoepidemiological claims data analysis. JAMA Neurol.

[16] Torres-Bondia F, Dakterzada F, Galván L, Buti M, Besanson G, Gill E (2020). Proton pump inhibitors and the risk of Alzheimer's disease and non-Alzheimer's dementias. Sci Rep.

[17] Hussain S, Singh A, Zameer S, Jamali MC, Baxi H, Rahman SO (2020). No association between proton pump inhibitors use and risk of dementia: evidence from a meta-analysis. J Gastroenterol Hepatol.

[18] Vogiatzoglou A, Smith AD, Nurk E, Drevon CA, Ueland PM, Vollset SE (2013). Cognitive function in an elderly population: interaction between vitamin B12 status, depression, and apolipoprotein E ε4: the Hordaland Homocysteine Study. Psychosom Med.

[19] O'Leary F, Allman-Farinelli M, Samman S (2012). Vitamin B^12^ status, cognitive decline and dementia: a systematic review of prospective cohort studies. Br J Nutr.

[20] Reynolds E (2006). Vitamin B^12^, folic acid, and the nervous system. Lancet Neurol.

[21] Cheng FC, Ho YF, Hung LC, Chen CF, Tsai TH (2002). Determination and pharmacokinetic profile of omeprazole in rat blood, brain and bile by microdialysis and high-performance liquid chromatography. J Chromatogr A.

[22] Rojo LE, Alzate-Morales J, Saavedra IN, Davies P, Maccioni RB (2010). Selective interaction of lansoprazole and astemizole with tau polymers: potential new clinical use in diagnosis of Alzheimer's disease. J Alzheimers Dis.

[23] Badiola N, Alcalde V, Pujol A, Münter LM, Multhaup G, Lleó A (2013). The proton-pump inhibitor lansoprazole enhances amyloid beta production. PLoS One.

[24] Majumdar A, Cruz D, Asamoah N, Buxbaum A, Sohar I, Lobel P (2007). Activation of microglia acidifies lysosomes and leads to degradation of Alzheimer amyloid fibrils. Mol Biol Cell.

[25] Pillay CS, Elliott E, Dennison C (2002). Endolysosomal proteolysis and its regulation. Biochem J.

[26] Mattsson JP, Väänänen K, Wallmark B, Lorentzon P (1991). Omeprazole and bafilomycin, two proton pump inhibitors: differentiation of their effects on gastric, kidney and bone H(+)-translocating ATPases. Biochim Biophys Acta.

[27] Moayyedi P, Eikelboom JW, Bosch J, Connolly SJ, Dyal L, Shestakovska O (2019). Safety of proton pump inhibitors based on a large, multi-year, randomized trial of patients receiving rivaroxaban or aspirin. Gastroenterology.

[28] Lochhead P, Hagan K, Joshi AD, Khalili H, Nguyen LH, Grodstein F (2017). Association between proton pump inhibitor use and cognitive function in women. Gastroenterology.

[29] Wod M, Hallas J, Andersen K, Rodríguez LAG, Christensen K, Gaist D (2018). Lack of association between proton pump inhibitor use and cognitive decline. Clin Gastroenterol Hepatol.

[30] Hwang IC, Chang J, Park SM (2018). A nationwide population-based cohort study of dementia risk among acid suppressant users. Am J Geriatr Psychiatry.

[31] Park SK, Baek YH, Pratt N, Ellett LL, Shin JY (2018). The uncertainty of the association between proton pump inhibitor use and the risk of dementia: prescription sequence symmetry analysis using a Korean healthcare database between 2002 and 2013. Drug Saf.

[32] Welu J, Metzger J, Bebensee S, Ahrendt A, Vasek M (2019). Proton pump inhibitor use and risk of dementia in the veteran population. Fed Pract.

[33] Chen LY, Lin HJ, Wu WT, Chen YC, Chen CL, Kao J (2020). Clinical use of acid suppressants and risk of dementia in the elderly: a pharmaco-epidemiological cohort study. Int J Environ Res Public Health.

[34] Cooksey R, Kennedy J, Dennis MS, Escott-Price V, Lyons RA, Seaborne M (2020). Proton pump inhibitors and dementia risk: evidence from a cohort study using linked routinely collected national health data in Wales, UK. PLoS One.

[35] Goldstein FC, Steenland K, Zhao L, Wharton W, Levey AI, Hajjar I (2017). Proton pump inhibitors and risk of mild cognitive impairment and dementia. J Am Geriatr Soc.

[36] Park SK, Nam JH, Lee H, Chung H, Lee EK, Shin JY (2019). Beyond uncertainty: negative findings for the association between the use of proton pump inhibitors and risk of dementia. J Gastroenterol Hepatol.

[37] Huang ST, Tseng LY, Chen LK, Peng LN, Hsiao FY (2019). Does long-term proton pump inhibitor use increase risk of dementia? Not really! Results of the group-based trajectory analysis. Clin Pharmacol Ther.

[38] Wu CL, Lei WY, Wang JS, Lin CE, Chen CL, Wen SH (2020). Acid suppressants use and the risk of dementia: a population-based propensity score-matched cohort study. PLoS One.

[39] Imfeld P, Bodmer M, Jick SS, Meier CR (2018). Proton pump inhibitor use and risk of developing Alzheimer's disease or vascular dementia: a case-control analysis. Drug Saf.

[40] Taipale H, Tolppanen AM, Tiihonen M, Tanskanen A, Tiihonen J, Hartikainen S (2017). No association between proton pump inhibitor use and risk of Alzheimer's disease. Am J Gastroenterol.

[41] Booker A, Jacob LE, Rapp M, Bohlken J, Kostev K (2016). Risk factors for dementia diagnosis in German primary care practices. Int Psychogeriatr.

[42] Ma C, Shaheen AA, Congly SE, Andrews CN, Moayyedi P, Forbes N (2020). Interpreting reported risks associated with use of proton pump inhibitors: residual confounding in a 10-year analysis of national ambulatory data. Gastroenterology.

[43] Gray SL, Walker RL, Dublin S, Yu O, Bowles EJA, Anderson ML (2018). Proton pump inhibitor use and dementia risk: prospective population-based study. J Am Geriatr Soc.

[44] Zhang Y, Liang M, Sun C, Song EJ, Cheng C, Shi T (2020). Proton pump inhibitors use and dementia risk: a meta-analysis of cohort studies. Eur J Clin Pharmacol.

[45] Khan MA, Yuan Y, Iqbal U, Kamal S, Khan M, Khan Z (2020). No association linking short-term proton pump inhibitor use to dementia: systematic review and meta-analysis of observational studies. Am J Gastroenterol.

[46] Desai M, Nutalapati V, Srinivasan S, Fathallah J, Dasari C, Chandrasekhar VT (2020). Proton pump inhibitors do not increase the risk of dementia: a systematic review and meta-analysis of prospective studies. Dis Esophagus.

[47] Song YQ, Li Y, Zhang SL, Gao J, Feng SY (2019). Proton pump inhibitor use does not increase dementia and Alzheimer's disease risk: an updated meta-analysis of published studies involving 642305 patients. PLoS One.

[48] Zhang Y, Zhan J, Bao Q, Lu J, Tan L (2020). Possible dementia risk of proton pump inhibitors and H2 receptor blockers use in the treatment of Helicobacter pylori: a meta-analysis study. Med Hypotheses.

